# Long-Term Follow-Up in Patients with Large-Vessel Vasculitis Applying Extracranial and Transcranial Duplex Sonography

**DOI:** 10.3390/diagnostics16030455

**Published:** 2026-02-01

**Authors:** Johanna Härtl, Sebastian Lambrecht, Felix Hess, Achim Berthele, Silke Wunderlich, Enayatullah Baki

**Affiliations:** Department of Neurology, TUM Universitätsklinikum Klinikum Rechts der Isar, School of Medicine and Health, Technical University of Munich, Ismaningerstr. 22, 81675 München, Germany

**Keywords:** giant cell arteritis, Takayasu arteritis, duplex sonography follow-up

## Abstract

**Background:** Although large-vessel vasculitis (LVV) can affect both the anterior and posterior intracranial circulation, routine neurosonographic follow-up, including transcranial duplex sonography, has not been established. We aimed to characterize patients with giant cell arteritis (GCA) and Takayasu arteritis (TAK) regarding the detection of progressive or new-onset inflammatory vessel changes by using neurosonography, and to assess the impact on medical or interventional treatment strategies. **Methods:** We retrospectively identified all patients with LVV treated at our neurological department between January 2015 and October 2025 with at least one neurosonographic follow-up examination. Baseline and follow-up sonographic data, clinical characteristics, medical therapy, and interventional treatments were analyzed. **Results:** In total, 21 LVV patients (GCA, n = 16; TAK, n = 5) underwent sonographic follow-up (GCA: median 28 (2–106) months, 4.5 (2–33) sonographic assessments; TAK: 75 (33–255) months, 14 (4–60) sonographic assessments). Isolated or combined, progressive or new-onset intra- and extracranial arterial disease was detected in seven of the 16 GCA patients (43.8%), of whom three (18.8%) presented with ischemic stroke. Medical treatment was adapted in four progressive cases. In two patients, additional interventional treatment was performed. Among TAK, two of five (40%) patients showed progressive sonographic changes, with one patient experiencing an ischemic stroke requiring endovascular treatment for progressive common carotid artery stenosis and one patient showing asymptomatic intracranial ICA involvement. **Conclusions:** Progressive and symptomatic involvement of intracranial carotid and vertebral arteries is a frequent finding in patients with LVV. These changes can be effectively detected through comprehensive neurosonographic follow-up, including transcranial ultrasound assessment.

## 1. Introduction

In addition to its diagnostic value, vascular ultrasound is increasingly used to monitor disease activity and the treatment response in large-vessel vasculitis (LVV) [1,2,3,4,5,6]. For giant cell arteritis (GCA), ultrasound examination of the extracranial arteries is routinely considered to monitor treatment and to detect disease relapses since IL-6 antibody therapy obscures laboratory markers of inflammation. The Outcome Measures in Rheumatology (OMERACT) Large Vessel Vasculitis Ultrasound Working Group recently developed a standardized ultrasound score (OGUS) for GCA, designed to quantify disease activity and enable longitudinal monitoring [7]. The OGUS includes intima-media thickness (IMT) quotient measurements of the main branch of the superficial temporal artery, its frontal and parietal branches, and the axillary arteries, normalized to established reference values. Higher OGUS values may reflect greater inflammatory activity, potentially hold prognostic relevance, and indicate treatment failure [8]. Most current sonographic follow-up protocols for GCA rely on the OGUS or related IMT-based assessments, while only a few studies have additionally evaluated the extracranial carotid and vertebral arteries [1,2,3,9,10,11], and none have evaluated sonographic measurements of intracranial arteries. More recently, transorbital sonography measuring peak systolic velocity (PSV) of the central retinal artery has been applied, which may complement ophthalmologic evaluation and aid in the detection of ischemic complications in GCA [12,13].

Similarly, for Takayasu disease, the sonographic follow-up of brachiocephalic artery changes, with a focus on IMT measurements, neovascularization, and assessment of echogenicity, has proven valuable for long-term follow-up and monitoring of inflammatory vascular changes [14,15,16,17,18,19].

Neurosonographic follow-up examinations of intracranial arteries have not been systematically implemented in current monitoring protocols for either TAK or GCA. This gap persists despite intracranial arterial involvement being a recognized manifestation in both GCA and TAK. Intracranial vasculitis has been reported in approximately 7% of TAK patients. The cumulative risk of embolic or hemodynamic ischemic stroke in TAK with extra- and intracranial artery involvement is reported as 15–20% of patients [20,21,22,23]. In GCA, ischemic stroke occurs in about 3–7%, with emerging evidence suggesting that intracranial artery involvement may be more common than previously assumed: high-resolution black-blood magnetic resonance imaging (MRI) identified intracranial vessel wall alterations in 14.5% of GCA patients [24], and a recent systematic literature review of 102 studies summarized a total of 340 patients with reported intracranial vasculitis [25].

Currently, data on the frequency, temporal evolution, and potential therapeutic implications of intracranial vascular involvement as assessed by transcranial sonography remain scarce. Therefore, we conducted a retrospective analysis of patients with large-vessel vasculitis and serial sonographic follow-up at our comprehensive stroke center to evaluate the prevalence of neurosonographic changes and their impact on therapeutic decision-making in LVV.

## 2. Materials and Methods

### 2.1. Data Source, Patient Selection, and Cohort Development

In a retrospective approach, we identified all patients who were treated at our neurological department for giant cell arteritis or Takayasu arteritis and underwent at least one clinical and sonographic follow-up examination after baseline assessment between January 2015 and October 2025. All diagnoses were made in accordance with the ACR/EULAR criteria and confirmed by expert neurologists [26,27]. Sonographic follow-up was scheduled as indicated by clinical assessment. Clinical and sonographic data were retrieved from medical records.

### 2.2. Demographic, Clinical, and Sonographic Characteristics

Patient demographic and clinical characteristics included age at diagnosis and sex. We assessed the follow-up period and the number of sonographic examinations performed. Sonographic results were characterized at baseline by the presence of a halo sign in the temporal arteries (TA) and inflammatory involvement of the vertebral artery (VA), the subclavian artery (SA), the common carotid artery (CCA), the extracranial internal carotid artery (ICA), and intracranial arteries. New-onset inflammatory changes at follow-up sonography were assessed according to the anatomic site and time course of the disease. Medication and changes in treatment strategies, including medical and interventional procedures, were analyzed in patients with new-onset or progressive inflammatory vessel changes.

### 2.3. Sonographic Outcome Definitions

New-onset inflammatory artery disease was defined as the diagnosis of any newly detected vascular involvement during sonographic follow-up that was not reported at baseline sonographic assessment.

Progressive inflammatory arterial disease was defined as any significant deterioration of a previously documented vascular abnormality identified at baseline sonographic assessment, as demonstrated by increased severity at follow-up, such as progression of arterial stenosis.

### 2.4. Statistical Analysis

All statistical analyses were performed using “R” (version 1.4.1717; R Foundation for Statistical Computing, Vienna, Austria). Descriptive statistics were presented as frequencies with percentages for categorical variables, as means with standard deviations for normally distributed continuous variables, and as medians with ranges for ordinal or non-normally distributed variables as appropriate.

## 3. Results

### 3.1. Patient Cohort

**Figure 1:**
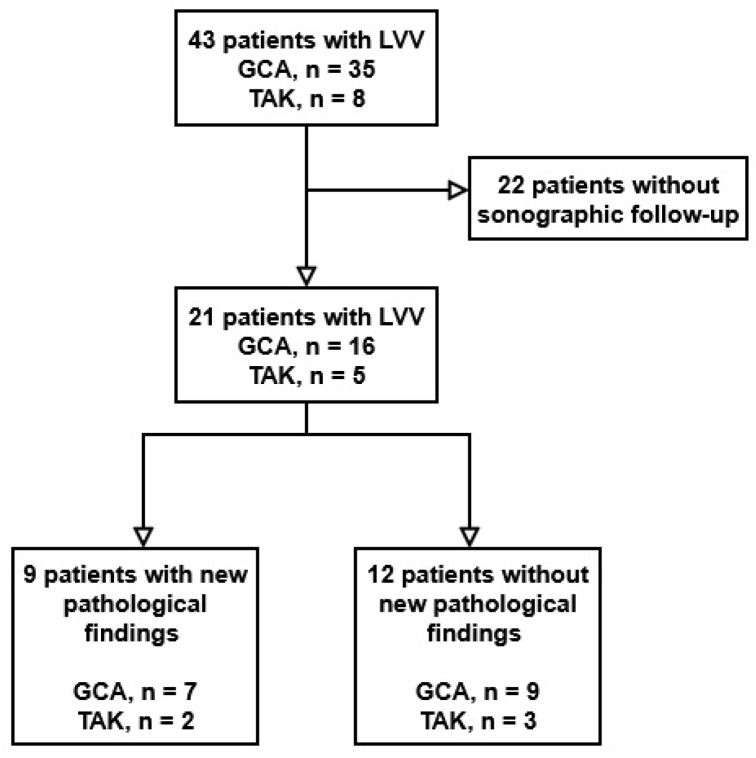
Study flow chart.

Overall, 43 patients were treated in our neurological department for GCA or TAK between January 2015 and October 2025. Twenty-two patients did not present for sonographic follow-up evaluation and were therefore excluded from the study. In total, we identified 21 patients who met the above-defined criteria. Of these, 16 patients were diagnosed with GCA and five patients with TAK. The follow-up period was a median of 28 (2–106) months, with a median of 4.5 (2–33) sonographic assessments for GCA and 75 (33–255) months with 14 (4–60) sonographic assessments for TAK. At the baseline sonographic assessment, three patients with GCA (18.8%) and two patients with TAK (40%) had intracranial involvement of vasculitis. The study flow chart is depicted in Figure 1. A comparison of baseline characteristics (age, sex, diagnosis) between the 22 patients excluded from the final analysis due to loss to follow-up and the 21 patients included in the final analysis is depicted in Supplementary Table S1.

### 3.2. Patients with New-Onset or Progressive Inflammatory Artery Disease in GCA

**Figure 2:**
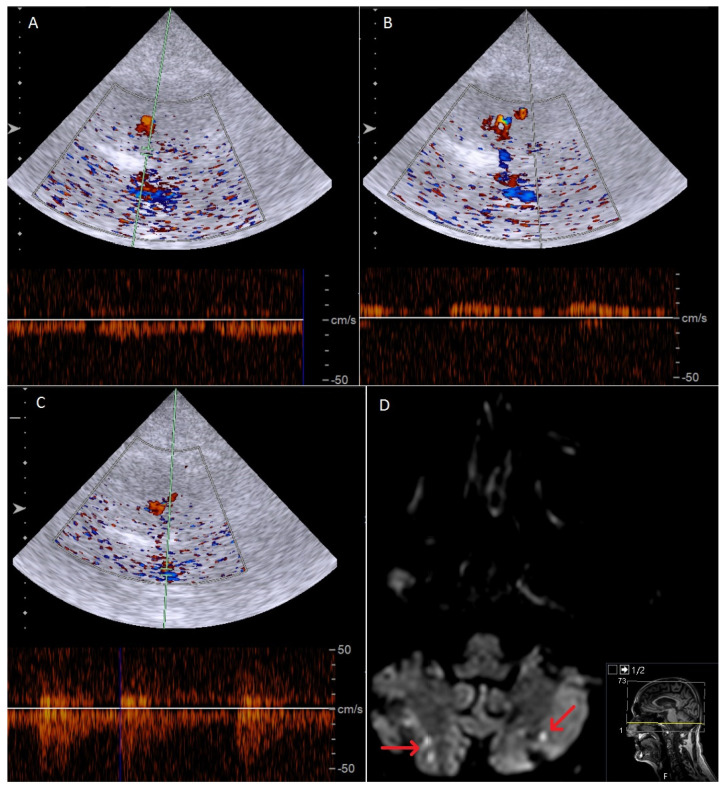
Exemplary case of a 72−year-old patient with GCA and images of a follow-up examination 4 months after the first diagnosis showing bilateral vertebral involvement. (**A**) Transnuchal duplex sonography showing highly reduced pulsatility of the right distal VA. (**B**) Retrograde perfused left distal VA. (**C**) Decreased blood flow in the basilar artery. (**D**) MRI scan (Diffusion Weighted Imaging sequence) showing new bilateral cerebellar infarctions (red arrows).

**Figure 3:**
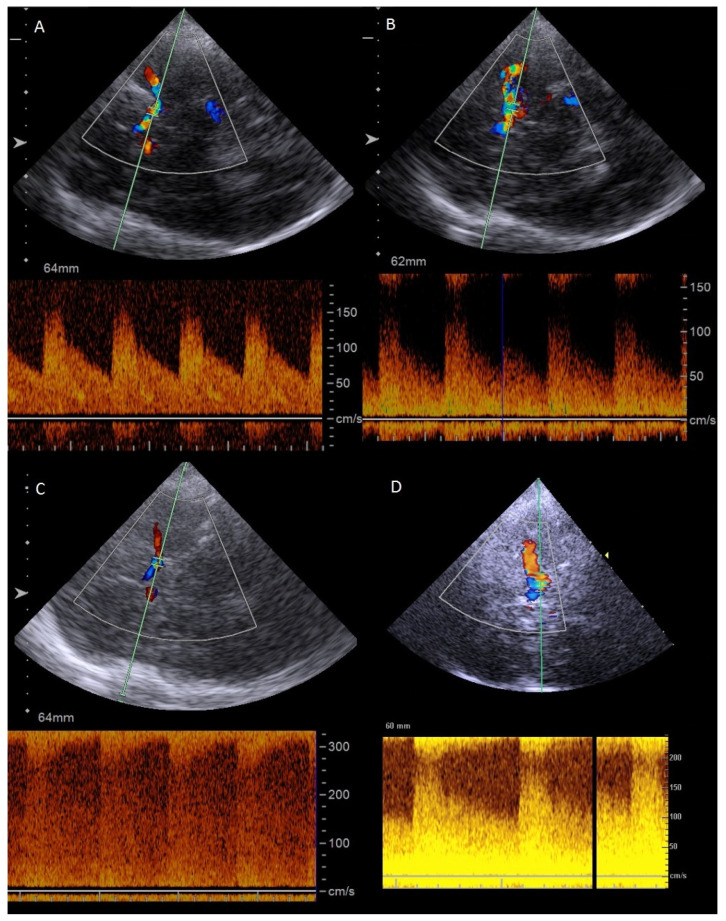
Exemplary case of a 64-year-old patient with GCA and transcranial sonographic images showing bilateral, distal ICA stenosis. (**A**,**B**) Baseline duplex sonography showing mildly increased blood flow velocity of the left (**A**) distal ICA and of the right (**B**) distal ICA. (**C**,**D**) Follow-up duplex sonography one month after baseline assessment showing highly increased blood flow velocity of the left (**C**) distal ICA and of the right (**D**) distal ICA.

In total, we detected new-onset or progressive inflammatory artery disease in seven of the 16 (43.8%) GCA patients. Patient characteristics are depicted in Table 1 and Table 2. Three patients (18.8%) presented with progressive halo sign in the TA; however, only one of these was symptomatic with headaches, and medical treatment was hence adjusted. We further observed one case of symptomatic new-onset intracranial anterior circulation disease, and two cases of combined symptomatic intracranial anterior and posterior artery disease, with one of these having combined extra- and intracranial VA involvement. All patients with new-onset or progressive intracranial inflammatory artery disease suffered ischemic complications and required adjustment of medical therapy. In two patients, additional interventional treatment of the anterior intracranial circulation was performed (stent angioplasty of the intracranial ICA, balloon dilatation of the intracranial ICA). Lastly, we observed one case of asymptomatic progressive extracranial VA involvement, which slightly regressed over a total follow-up of four months. Figure 2 illustrates the case of a 72-year-old GCA patient with intracranial VA involvement 4 months after the first diagnosis and bilateral cerebellar infarctions in the MRI scan. Figure 3 shows the case of a 64-year-old GCA patient with progression of bilateral, distal ICA stenosis.

### 3.3. Patients with New-Onset or Progressive Inflammatory Artery Disease in TAK

**Figure 4:**
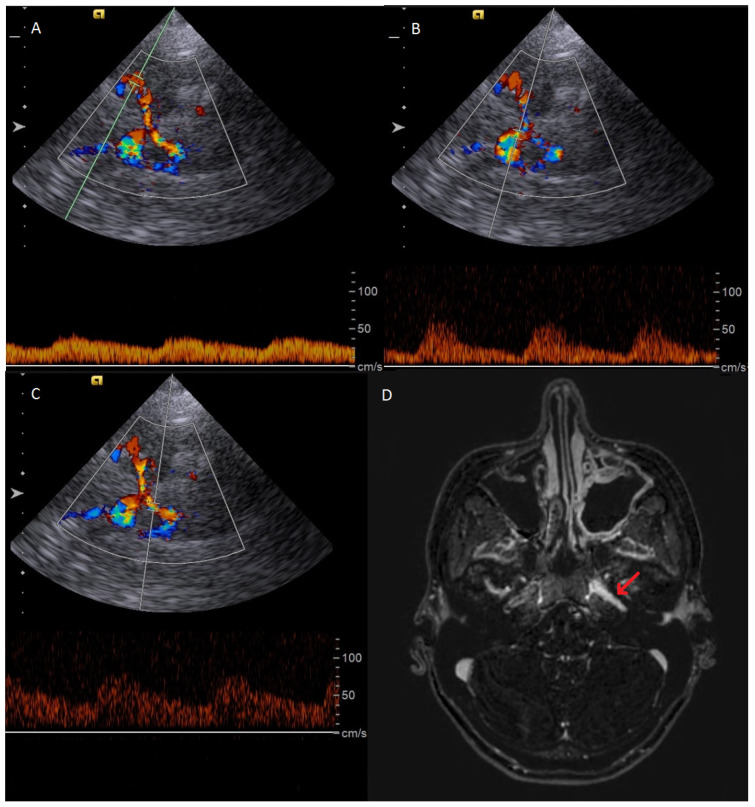
Exemplary case of a patient with TAK and images of a follow-up examination 5 years after the first diagnosis showing signs of high-grade stenosis of the left distal ICA. (**A**) Transcranial duplex sonography showing reduced pulsatility of the left distal MCA. (**B**) Retrograde perfused left ACA. (**C**) Cross-flow circulation from the left PCA to the left MCA through the posterior communicating artery (**D**) MRI scan (subtraction T1-weighted 3D TRA sequence) confirming high-grade stenosis and vascular inflammation of the left distal ICA (red arrow).

In total, we detected new-onset or progressive inflammatory artery disease in two of five TAK patients. Patient characteristics are depicted in Table 1 and Table 2. One patient presented with asymptomatic intracranial involvement of the ICA, but we refrained from adjusting medical therapy. The sonographic and MR images of this patient are depicted in Figure 4. One patient presented with ischemic stroke due to progressive CCA involvement, and stent angioplasty of the CCA was performed.

## 4. Discussion

We present the first study to demonstrate the value of transcranial neurosonography for detecting new-onset or progressive intracranial inflammatory vessel involvement during routine baseline and follow-up visits for LVV. To date, a comprehensive neurosonographic approach, including both transcranial and transnuchal sonography, has not been integrated into proposed study designs for sonographic follow-up or disease monitoring in GCA or TAK.

In GCA, current sonographic follow-up protocols primarily emphasize the measurement of IMT of the temporal and axillary arteries, as captured by composite scoring systems such as the OGUS, the halo count, the halo score, and the Southend halo score [2,3]. The clinical utility of these standardized ultrasound-based scoring systems in follow-up sonographic assessment has been supported by several studies. Inter alia, Kirby et al. demonstrated that the use of the OGUS, halo score, and halo count facilitated reproducible and standardized disease monitoring in clinical practice [9]. Ponte et al. reported that sonographic changes in the temporal and axillary arteries—particularly regression of the halo sign and a reduction in IMT—can occur rapidly in response to effective glucocorticoid therapy [28].

The assessment of further supraaortic branches—including the VA, SA, brachial artery, CCA, and ICA—has been incorporated into sonographic follow-up protocols, in addition to the scoring systems described above. Thus far, the examined arteries and their reported treatment responses vary between study protocols. For example, Seitz et al. reported a decline in the IMT of the SA, in addition to reductions in the axillary arteries and TA, after treatment induction with methylprednisolone pulse therapy followed by tocilizumab [29]. The treatment effect of tocilizumab, as assessed by sonographic IMT measurements, appeared later in the axillary and subclavian arteries than in the temporal artery. Hansen et al., in a prospective study of 48 therapy-naïve GCA patients, noted a rapid decrease in IMT in the temporal and axillary arteries within ten days of glucocorticoid initiation, in contrast to the slower changes in other supraaortic arteries, including the CCA and brachial arteries [10]. Similarly, Ford et al. demonstrated a rapid regression of inflammatory changes in the TA, whereas involvement of the SA and axillary arteries tended to persist on sonographic follow-up in a retrospective evaluation of 42 patients with a median follow-up of 5.1 months [30]. Haaversen et al. prospectively evaluated 132 GCA patients for halo signs in the temporal and facial arteries as well as for IMT changes in supraaortic vessels, including the CCA, VA, and SA, and reported moderate sensitivity and specificity for detecting clinical relapse as defined by the EULAR definitions of key symptoms and clinical findings suggestive of active disease [11]. Nielsen et al. performed a prospective clinical and sonographic follow-up of 47 patients at 8 weeks, 24 weeks, and 15 months, demonstrating that IMT measurements of the TA—particularly those included in the OGUS score—showed a moderate-to-strong correlation with disease activity. In contrast, large-vessel involvement, as assessed by IMT of the CCA, was associated with a poor treatment response [31]. Aschwanden et al. conducted a 24-month longitudinal follow-up of 42 patients, with a median of two sonographic examinations per patient. They found that regression of IMT in large arteries—specifically the CCA, ICA, external carotid artery, VA, SA, axillary artery, popliteal artery, and superficial femoral artery—was substantially less common than IMT reduction in the TA [32]. Schäfer et al. confirmed significant decreases in the total number of affected arteries, i.e., the CCA and VA, and in OGUS values, in 36 newly diagnosed patients who completed a 12-month follow-up period with regular sonographic assessments after treatment initiation [1]. In contrast to some of the previously described studies, in these patients, the mean IMT of the axillary artery normalized before the common TA.

For TAK, sonographic follow-up protocols mostly include IMT measurement of the CCA, SA, and VA, with an additional focus on vessel wall echogenicity. It has been reported that hypoechogenic vessel walls are commonly more prevalent in acute or new-onset inflammatory diseases, with possible long-term progression to hyperechogenic vessel wall changes accompanied by fibrotic stripes [14,18,33,34]. Thus far, various scores, including the above-mentioned parameters, have been proposed for differentiating active disease. Firstly, combining echogenicity and IMT measurements of the CCA, SA, and axillary artery, the Takayasu Ultrasound Index has been validated in the follow-up assessment of TAK [18]. Similarly, the ultrasonographic activity score (ULTRAS) has recently been proposed, which includes wall thickness and semi-quantitative echogenicity scores in patients with CCA involvement. Additionally, neovascularization, as detected by contrast-enhanced ultrasound and the presence of intramural arteries, has been described, indicating secondary inflammatory changes in TAK, which may be sonographically detectable in follow-up assessments [15,16,18]. Nevertheless, sonography has not been established for routine diagnostic follow-up in TAK [17,19]. If performed, the frequency of imaging follow-up in TAK has not been specified. A recent retrospective multicenter study favors patient-specific and individualized periods, as radiological progress was unlikely in the absence of clinical activity, but in the case of its detection, seemed highly relevant for medical treatment [19].

Collectively, these findings highlight the potential value of sonographic parameters as biomarkers of disease activity and treatment response in LVV. Since the pattern of vessels is highly variable, we propose adding transcranial sonography to the standard LVV work-up. Although inflammatory involvement of intracranial arteries has been described in both TAK and GCA [20,21,22,23,24,25], transcranial sonography has not been implemented in sonographic protocols thus far. In our patient population, baseline as well as new-onset or progressive intracranial artery involvement was relatively common in both GCA and TAK, and was well detected by a complete sonographic examination. Hence, we argue that the expansion of sonographic protocols, incorporating transcranial and transnuchal approaches in addition to a complete assessment of VA, CCA, and ICA, provides a more comprehensive evaluation of vascular inflammation in both baseline and follow-up assessments, and may identify patients at risk of ischemic complications.

Recent evidence suggests that transorbital sonography, with measurement of the peak systolic velocity (PSV) of the central retinal artery, may provide a tool for the assessment of intracranial vascular involvement in GCA [12,13]. Since the PSV of the central retinal artery can be directly affected by intracranial ICA pathology, this finding highlights the clinical relevance and frequency of intracranial artery involvement in GCA, and thereby the importance of additionally performing a comprehensive neurosonographic work-up that includes a complete transcranial assessment.

Data on the prevalence of extra- and intracranial involvement of the VA in GCA remain heterogeneous. Reported frequencies vary considerably depending on the characteristics of the studied cohort, particularly in patients presenting with GCA-associated stroke, and on the imaging modality employed [19,20,21]. For example, Penet et al. identified VA involvement in 2.4% of patients when assessed by sonography, whereas positron emission tomography (PET) revealed involvement in 22% [22]. Intracranial VA involvement has been reported in 83.9% of patients with intracranial GCA and GCA-related stroke, predominantly affecting the V3 and V4 segments before the origin of the posterior inferior cerebellar artery (PICA), and typically exhibiting the characteristic “slope sign” [19]. At baseline, we detected extra- or intracranial involvement of the VA in 50% of patients within our neurological cohort.

In our study, intracranial ICA involvement at baseline was identified in 18.8% of GCA patients. More recent evidence suggests that intracranial large-vessel anterior arteritis may be more common than previously recognized, predominantly detected by high-resolution MR imaging. Guggenberger et al. reported intracranial ICA involvement in 10.9% of patients using MR imaging with black-blood sequences and MR angiography [24]. Other MR-based studies have demonstrated intracranial ICA involvement in up to 50% of patients with GCA [35]. While vessel wall imaging is not feasible in transcranial ultrasound, the resulting hemodynamic ICA stenoses can be reliably identified, which may thus serve as a practical modality for longitudinal follow-up.

The incidence of GCA-related stroke remains controversial, with reported rates ranging from 1.5% to 16%. Strokes have been reported to predominantly affect the vertebrobasilar circulation and are more frequently observed in male patients [36,37,38,39]. In our cohort, GCA-related stroke occurred in 18.8% of patients, all of whom exhibited sonographically detected new-onset or progressive inflammatory involvement of intracranial arteries and presented with intracranial VA stenosis at baseline as a possible risk factor for GCA-related stroke [39]. Of note, and contrary to the previously discussed literature, there was, even though limited by the restricted patient numbers, no evident increase in the occurrence of vertebro-basilar ischemic stroke in our patient cohort.

The finding of a progressive halo sign without clinical relevance in two GCA patients strengthens the importance of a standardized assessment using the proposed OGUS score, which was not routinely assessed during the study period [1,7,8].

The key limitation of our study is the relatively small sample size, which restricts the extent to which the findings can be generalized. However, our findings provide valuable clinical insights into a rare disease for which data are overall scarce, helping to expand the limited evidence base and create a potential blueprint for future prospective multicenter studies. Secondly, the retrospective design of the study represents another important limitation, as our analyses depend on clinical data from medical records that were not primarily collected for research purposes. For GCA, the above-described therapeutic uncertainties regarding both the localization and duration of inflammatory vessel wall changes following treatment initiation have thus far prevented sonography from being included in international EULAR recommendations on the clinical follow-up of patients without suspected disease relapse [5]. This lack of consensus on a standardized sonographic follow-up may account for the high rate of patients without sonographic follow-up, resulting in the discussed restricted numbers of included patients and possible selection and attrition bias. In our study, approximately half of the patients did not return for sonographic follow-up, representing a relatively high dropout rate. Due to the retrospective design, the reasons for non-attendance are unknown, and the resulting attrition bias limits the interpretation of the true prevalence of newly identified pathologies. One possible explanation for non-attendance at follow-up could be that some patients were clinically stable and asymptomatic, and therefore did not perceive a need for further evaluation. Thus, the pathological findings observed in the remaining cohort may appear more pronounced, giving a misleading impression of the true prevalence of pathological findings in sonographic follow-up of LVV patients.

Overall, this study demonstrates the feasibility and potential diagnostic yield of transcranial sonographic monitoring. However, it is not suitable to define a standardized monitoring protocol, mainly owing to its retrospective design and limited sample size. Notably, this is the first study to highlight these considerations, delineating the primary motivation for a prospective, multicenter trial with clear, protocol-defined time points and parameters for clinical and sonographic assessments. A large prospective trial would enable systematic evaluation of disease progression and the utility of sonographic monitoring, while minimizing bias and enhancing generalizability.

## 5. Conclusions

In conclusion, this study highlights the potential medical implications of comprehensive neurosonographic assessment in the follow-up of patients with GCA and TAK. The addition of transcranial and transnuchal sonography to the established protocols may help to detect progressive or new-onset inflammatory artery disease in known LVV, thereby influencing medical and interventional decision-making. Incorporating these modalities into routine follow-up examinations of patients with LVV could facilitate earlier recognition of clinically silent progression and hence identify patients at risk for ischemic complications. The central innovative aspect of this study lies in the inclusion of transcranial sonography, representing, to the best of our knowledge, the first systematic evaluation of this modality in patients with large-vessel vasculitis. By extending ultrasound assessment beyond extracranial vessels, this approach supports a more comprehensive, non-invasive diagnostic strategy that may contribute to future updates and refinements of EULAR recommendations for the imaging-based diagnosis and monitoring of LVV. Further prospective studies are warranted to validate these observations and to clarify their impact on patient outcomes.

## Figures and Tables

**Table 1:** Baseline demographic, clinical, and sonographic characteristics. SD = standard deviation, n = number of patients, IA = intracranial arteries, VA = vertebral artery, CCA = common carotid artery, ICA = internal carotid artery, SA = subclavian artery.

Patient Characteristics			
Patients		GCA	TAK
	Number of patients included	16	5
	Age (years), mean (±SD)	72.8 ± 7.4	37.6 ± 8.4
	Gender		
	Female *n* (%)	9 (56.3%)	3 (60%)
	Male *n* (%)	7 (43.8%)	2 (40%)
	Follow-up period (months), median (range)	28 (2–106)	75 (33–255)
	Number of sonographic assessments during follow-up, median (range)	4.5 (2–33)	14 (4–60)
Baseline sonography	Halo sign TA, *n* (%)	13 (81.3%)	-
	Involvement of VA, *n* (%)	8 (50.0%)	2 (40%)
	Involvement of SA, *n* (%)	-	5 (100%)
	Involvement of CCA, *n* (%)	-	2 (40%)
	Involvement of extracranial ICA, *n* (%)	-	2 (40%)
	Involvement of anterior circulation IA, *n* (%)	3 (18.8%)	2 (40%)
New or progressive artery involvement detected in follow-up sonography	Total, *n* (%)	7 (43.8%)	2 (40%)
	Symptomatic progress, *n* (%)	4 (25.0%)	1 (20%)
	Progress or new-onset involvement of VA, *n* (%)	3 (18.8%)	-
	Progress or new-onset involvement of anterior circulation IA, *n* (%)	3 (18.8%)	1 (20%)
	Progress or new-onset involvement of CCA, *n* (%)	-	1 (20%)
	Change of medical treatment following progress, *n* (%)	4 (25.0%)	-
	Interventional treatment following progress, *n* (%)	2 (12.5%)	1 (20%)

**Table 2:** Clinical and sonographical findings in patients with progressive inflammatory artery disease and their medical impact. GCA = giant cell arteritis, TAK = Takayasu arteritis, f = female, m = male, TA = temporal artery, VA = vertebral artery, ICA = internal carotid artery, CCA = common carotid artery, SA = subclavian artery, MTX = methotrexate, CYC = cyclophosphamide, TOC = tocilizumab, MPS = methylprednisolone.

Clinical and Sonographic Findings in Patients with Progressive Inflammatory Artery Involvement in LVV and Their Therapeutic Impact											
Clinical Information						Progressive Inflammatory Artery Disease During Sonographic Follow-Up			Medical and Interventional Treatment Following Progress		
	Age	Sex	Follow-up Period (Months)	Number of Sonographic Examinations	Baseline Pathology	Time from First Diagnosis (Months)	Symptomatic Progress	Sonographic Findings	Baseline	Change	Interventional Treatment
GCA	68	m	105	20	Halo TA Extracranial VA stenosis	36	-	Progressive halo sign TA	Prednisolone + MTX	-	-
GCA	67	f	55	19	Occlusion intracranial right VA	3	Recurrent left-hemispheric strokes	High-grade stenosis of both ICAs (petrous segment) Stenosis MCA left	Prednisolone	MPS pulse (1000 mg for 5 days), MTX	Stent angioplasty of the left intracranial ICA
GCA	64	m	30	33	Halo TA Intracranial right VA stenosis Intracranial, bilateral ICA stenosis	1	Recurrent bihemispheric strokes	Progressive stenosis ICA and MCA left Progressive stenosis VA right extracranial and both intracranial VA	Prednisolone	MTX, TOC, CYC	Balloon dilatation of both ICAs
GCA	83	m	60	3	Halo TA	41	-	Progressive halo TA	Prednisolone + TOC	-	-
GCA	80	f	4	4	Halo TA Extracranial VA stenosis	1	-	Progressive extracranial, right-side VA stenosis	Prednisolone + MTX	-	-
GCA	72	m	23	6	Halo TA Intracranial VA stenosis	4	Recurrent vertebro-basilar strokes	Intracranial, bilateral VA occlusion Intracranial, bilateral ICA stenosis	Prednisolone + TOC	MPS pulse (1000 mg for 5 days), CYC, MTX	-
GCA	80	m	12	2	Halo TA	5	Headache	Progressive halo TA	Prednisolone + TOC	Prednisolone dose increase	-
TAK	26	f	75	14	VA, SA, CCA, ICA (extracranial)	57	-	Intracranial left-side ICA stenosis	Prednisolone + Certolizumab	-	-
TAK	33	f	254	60	SA, ACC	95	Right-hemispheric stroke	Progressive right-side CCA stenosis	Prednisolone + MTX	-	Stent angioplasty right CCA

## Data Availability

The data that support the findings of this study are available upon reasonable request from the corresponding author.

## Supplementary Materials

The following supporting information can be downloaded at: https://www.mdpi.com/article/10.3390/diagnostics16030455/s1, Table S1: Comparison of baseline characteristics of patients with GCA and TAK included and excluded from final analysis.

## Abbreviations

The following abbreviations are used in this manuscript:

ACA	anterior cerebral artery
BA	basilar artery
CCA	common carotid artery
GCA	giant cell arteritis
ICA	internal carotid artery
IMT	intima-media thickness
LVV	large-vessel vasculitis
MCA	middle cerebral artery
MRI	magnetic resonance imaging
OGUS	giant cell arteritis ultrasonography score
PCA	posterior cerebral artery
PSV	peak systolic velocity
SA	subclavian artery
TA	temporal artery
TAK	Takayasu arteritis
TCD	transcranial duplex sonography
VA	vertebral artery
