# Supplementary material for: Long-Term Follow-Up in Patients with Large-Vessel Vasculitis Applying Extracranial and Transcranial Duplex Sonography
Source: Diagnostics (Basel). 2026 Feb 1;16(3):455. doi: 10.3390/diagnostics16030455 (PMC12896947; doi:10.3390/diagnostics16030455)
Supplement: Supplementary file 1 [file diagnostics-16-00455-s001.zip › diagnostics-4077059-supplementary.pdf]

	GCA – included	GCA – excluded
Number of patients	16	19
Age (years), mean (±SD)	72.8 ± 7.4	77.0 ± 6.8
Gender		
Female, n (%)	9 (56.3%)	10 (52.6%)
Male, n (%)	7 (43.8%)	9 (47.3%)
	**TAK – included**	**TAK – excluded**
Number of patients	5	3
Age (years), mean (±SD)	37.6 ± 8.4	33 ± 16.6
Gender		
Female, n (%)	3 (60 %)	2 (66%)
Male, n (%)	2 (40 %)	1 (33%)

Supplementary Table S1: Comparison of baseline characteristics of patients with GCA and TAK included and excluded from final analysis
